# Optimal lymph node dissection for gastric cancer: a narrative review

**DOI:** 10.1186/s12957-024-03388-4

**Published:** 2024-04-23

**Authors:** Raphaël Nico, Julie Veziant, Amélie Chau, Clarisse Eveno, Guillaume Piessen

**Affiliations:** 1 Department of Digestive and Oncological Surgery, University Lille, Claude Huriez University Hospital, Lille, 59000 France; 2 CNRS, Inserm, UMR9020-U1277-CANTHER-Cancer, University Lille, CHU Lille, Lille, 59000 France; 3 FREGAT Network, Claude Huriez University Hospital, Lille, 59000 France; 4 Department of Digestive Surgery, Hénin-Beaumont Hospital, Hauts-de-France, France; 5 Rue Michel Polonowski, Lille Cedex, 59037 France

**Keywords:** Gastric cancer, Lymph node dissection, Guidelines, Worldwide

## Abstract

The management of gastric cancer has long been debated, particularly the extent of lymph node (LN) dissection required during curative surgery. LN invasion stands out as the most critical prognostic factor in gastric cancer. Historically, Japanese academic societies were the pioneers in defining a classification system for regional gastric LN stations, numbering them from 1 to 16. This classification was later used to differentiate between different types of LN dissection, such as D1, D2 and D3. However, these definitions were often considered too complex to be universally adopted, resulting in wide variations in recommendations from one country to another and making it difficult to compare published studies. In addition, the optimal extent of LN dissection remains uncertain, with initially recommended dissections being extensive but associated with significant morbidity without a clear survival benefit. The aim of this review is to make a case for extending LN dissection based on the existing literature, which includes a comprehensive examination of the current definitions of lymphadenectomy and an analysis of the results of all randomised controlled trials evaluating morbidity, mortality and long-term survival associated with different types of LN dissection. Finally, we provide a summary of the various recommendations issued by organizations such as the Japanese Gastric Research Association, the National Comprehensive Cancer Network, the European Society for Medical Oncology, and the French National Thesaurus of Digestive Oncology.

## Background

Lymph node (LN) invasion is the primary prognostic factor in gastric cancer, even after neoadjuvant chemotherapy [1]. Systematized and extensive lymphadenectomy requires anatomopathological expertise. The current AJCC/UICC TNM classification (8th edition) recommends the removal of at least 15 lymph nodes for reliable staging [2]. Some studies have demonstrated that a higher number of examined LNs correlates with improved post-gastrectomy survival rates in T1-3N0-1 gastric cancer [3]. The five-year survival rate is 70–84% in cases with no LN metastases (N0), 30% in cases with perigastric LN metastases (N1) and 5% in cases with regional LN metastases (N2) [4, 5]. Over the past forty years, the extent of LN dissection has been controversial. Historically, Asian authors have advocated for extensive LN dissection, highlighting their excellent long-term oncological outcomes, often superior to those observed in the West, albeit with low levels of evidence. The results of recent randomized controlled trials in Asia have led to a reduction in the recommended extent of LN dissection [6, 7], with no apparent adverse effect on survival. In Western countries, two early Dutch [8] and MRC [9] trials (D1 versus D2) and a more recent Italian trial (D1 versus D2 without systematic splenopancreatectomy (SPC)) [10] have been conducted. These three trials failed to demonstrate a survival advantage of D2 dissection in terms of overall survival (OS) in the overall population [8–10]. In the first two trials, D2 dissection was associated with excess morbidity and mortality associated with splenectomy or pancreatectomy [8, 9], which was not seen in the Italian trial [11]. However, in the Italian trial, the authors noted a positive trend in specific survival for patients with T2-T4 or N + tumours in favour of D2 without systematic SPC dissection [10], confirmed by the 15-year results published in 2021 [12]. Furthermore, long-term follow-up (15 years) in the Dutch trial showed fewer locoregional recurrences and gastric cancer-related deaths with D2 without systematic SPC dissection [13]. Since 2016, European guidelines recommend that patients undergo modified D2 without systematic SPC dissection in high-volume centres with surgical expertise [14]. Thus, differences in surgical practice between the West and Asia have diminished and procedures are becoming increasingly standardized through simplified definitions. The aim of this review is to develop the argument for extending LN dissection in the light of the data in the literature.

## Lymph node classification and lymphadenectomy definitions

### History

**Fig. 1:**
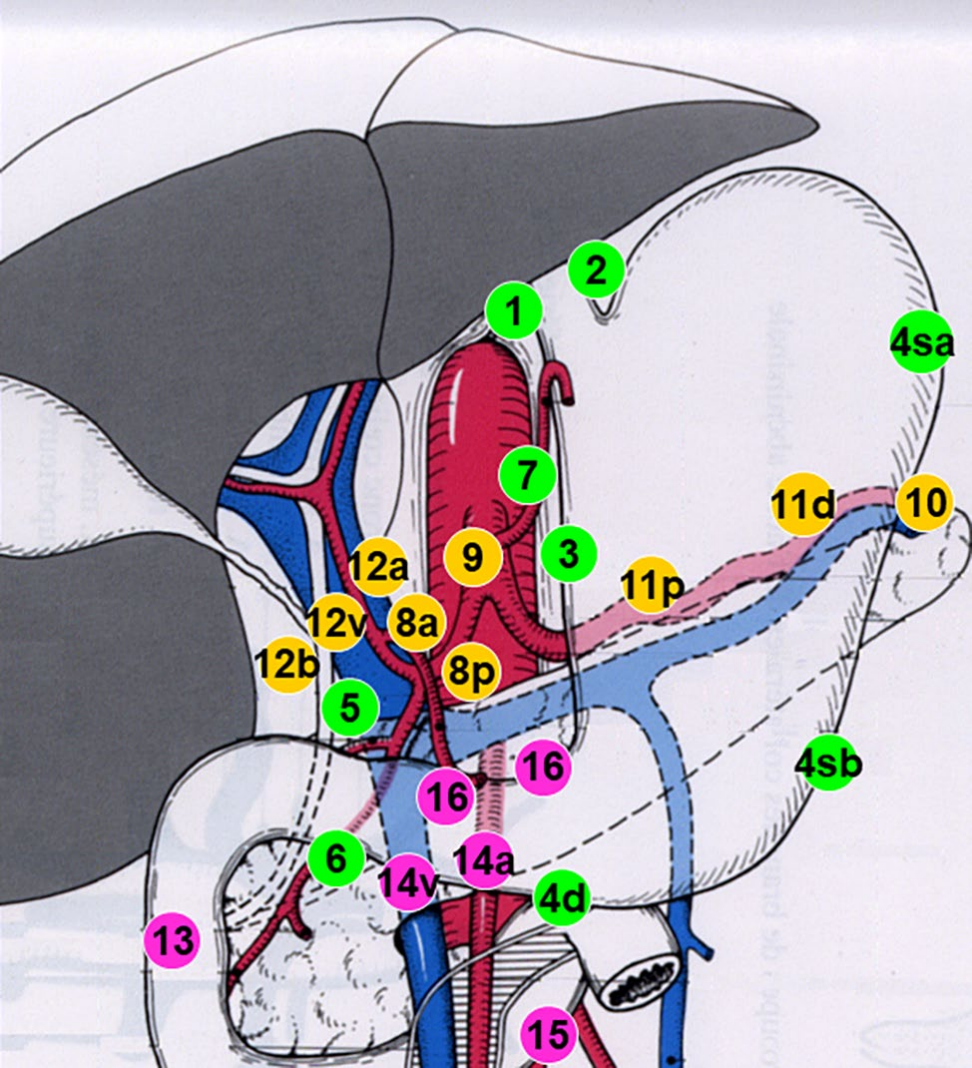
Lymphatic drainage diagram of the stomach according to the first classification of the Japanese Research Society for Gastric Cancer (JRSGC), adapted from [15]. First region: *Group 1*: Right paracardium *Group 2*: Left paracardial *Group 3*: Gastric lesser curvature *Group 4*: Gastric greater curvature divided into 4sa: short vessels 4sb: Left gastroepiploic artery 4d: Right gastroepiploic artery *Group 5*: Suprapyloric *Group 6*: Infrapyloric. Second region: *Group 7*: Left gastric artery *Group 8*: Common hepatic artery divided into 8a: Anterior 8p: Posterior *Group 9*: Celiac trunk *Group 10*: Splenic hilum *Group 11*: Splenic artery divided into 11p: Proximal 11d: Distal *Group 12*: Pedicle of liver divided into 12a: Artery 12b: bile duct 12v: Portal vein. Third region: *Group 13*: Retropancreatic *Group 14*: Superior mesenteric artery and vein, divided into 14v: Venous 14a: Arterial *Group 15*: Middle colic artery *Group 16*: Paraaortic divided into 16a1: Hiatus of the oesophagus 16a2: From the celiac trunk to the renal vein 16b1: From the renal vein to the inferior mesenteric artery 16b2: From the inferior mesenteric artery to the aortic bifurcation. Groups 19, 20, 110, 111 (which are not shown) correspond to lower mediastinal nodes to be resected in gastric tumours invading the oesophagus and are considered local regional nodes. They are not described in detail here

The Japanese Research Society for Gastric Cancer (JRSGC) defined and published the general rules for gastric cancer surgery as early as 1973, and in 1981 these rules were published in English, assigning individual numbers to each LN group (Fig. 1). This classification was widely disseminated and adopted in different countries. The regional gastric LN were thus divided into 16 groups [15]. Initially, LN stations in the gastric drainage area were classified into three groups (or four in some editions) based on the anatomical location of the station relative to the location of the primary tumour. These numbers were also used to express the grade of LN metastasis (N1-3) and the extent of lymphadenectomy (D1-3). This rule has remained consistent throughout the history of the JRSGC, although the details of the classification of LN groups have been modified with each new edition. For example, LN station n°14v was initially included in the D2 dissection for distal tumours but was subsequently excluded from the 3rd edition [16, 17].

The definitions established by Japanese academic societies were considered too complex to be universally adopted with accuracy. Firstly, tumour location may not have been accurately classified by surgeons/pathologists, resulting in the dissection of incorrect LN groups. Also, outside of clinical trials, the terms “D1-3” have not always been used accurately. Finally, outside of Japan, many believed that the nodes in the first group were perigastric nodes (1 to 6), those in the second group were along the celiac artery and its branches (7 to 11), and those in the third group were numbered from 12 to 16, defining D1, D2, and D3 dissections, respectively. Since 2010, the definition of lymphadenectomy has been significantly simplified: LN stations dissected in D1, D1 + and D2 are defined according to the type of gastrectomy, regardless of tumour location. D3 dissection is no longer defined, as the rationale for this recommendation of super-extended surgery beyond D2 dissection has been lost due to the negative results of the JCOG 9501 trial [16, 17].

### Definitions of D1 and D2 lymph node dissection

Table 1 summarizes the LN groups involved in D1 and D2 LN dissection based on the types of recommendations [18–20]. Since 2010, the Japanese Gastric Cancer Association (JGCA) has defined the type of dissection based on the type of gastrectomy [16, 17, 20], and LN group n°7 has been included in D1 dissection regardless of the type of gastrectomy [16]. Thus, in total gastrectomy, D1 dissection includes LN n° 1, 2, 3, 4, 5, 6, 7, while D2 dissection includes LN n°1, 2, 3, 4, 5, 6, 7, 8a, 9, 10, 11p, 11d, 12a [20]. In distal gastrectomy, D1 dissection includes LN n°1, 3, 4sb, 4d, 5, 6, 7, while D2 dissection includes LN n°1, 3, 4sb, 4d, 5, 6, 7, 8a, 9, 11p, 12a [20] (**Table I**). It is worth noting the existence of a recommended D1 + dissection in cases of cT1N0 tumours > 1.5 cm in diameter or poorly differentiated, which is a D1 dissection extended to LN n°8a, 9 and, in case of total gastrectomy, to station n°11p.

**Table 1:** Node groups to be resected for D1 and D2 lymphadenectomy according to various recommendations

Ref.	Country	Type of gastrectomy	D1 lymphadenectomy	Type of gastrectomy	D2 lymphadenectomy
ESMO, 2022 [18]	Europe	NP	N°1, 2, 3, 4, 5, 6, 7	NP	N°1, 2, 3, 4, 5, 6, 7, 8, 9, 11
NCCN, 2019 [19]	America	NP	N°1, 2, 3, 4, 5, 6	NP	N°1, 2, 3, 4, 5, 6, 7, 8, 9, 10, 11
JGCA, 2021 [20]	Japan	Total	N°1, 2, 3, 4, 5, 6, 7	Total	N°1, 2, 3, 4, 5, 6, 7, 8a, 9, 10, 11p, 11d, 12a
		Distal	N°1, 3, 4sb, 4d, 5, 6, 7	Distal	N°1, 3, 4sb, 4d, 5, 6, 7, 8a, 9, 11p, 12a

ESMO : European Society of Medical Oncology, NCCN : National Comprehensive Cancer Network, JGCA : Japanese Gastric Cancer Association, NP : not precised

The European Society of Medical Oncology (ESMO) and National Comprehensive Cancer Network (NCCN) guidelines have not yet established a clear relationship between the type of gastrectomy and the extent of LN dissection [18, 19]. According to the 2022 ESMO guidelines, D1 LN dissection includes perigastric LN n°1, 2, 3, 4, 5, 6 and the left gastric LN (n°7), which was included in D2 dissection in the 2016 recommendations. D2 LN dissection includes LN n°1, 2, 3, 4, 5, 6, 7, 8, 9, 11 [18]. Thus, the European guidelines have moved closer to the Japanese definitions by including group 7 in D1 dissection. According to the 2019 NCCN guidelines, D1 LN dissection includes LN n°1, 2, 3, 4, 5, 6, whereas D2 dissection includes dissection of LN n°1, 2, 3, 4, 5, 6, 7, 8, 9, 10, 11. These guidelines were updated in 2022, but this update only concerned chemotherapy [19]. In France, “D1.5” LN dissection is defined according to the 2009 recommendations of the SFCD (French Society of Digestive Surgery) [21] and to the 2022 recommendations of the TNCD (French National Thesaurus of Digestive Oncology) [22] as D1 dissection plus groups 7, 8, 9 and, in the case of proximal gastrectomy, the addition of splenic node 11 dissection without splenectomy. Groups 12a and 10 are not specifically mentioned. These discrepancies between different classifications make it difficult to analyze the literature. An ongoing survey within the European branch of the International Gastric Cancer Association (IGCC) is investigating the interpretation of these definitions.

### Definitions of D2+, D3 and D4 lymph node dissections

As mentioned above, these definitions have evolved over time, making it difficult to navigate the literature. The use of terms such as D2+, D3 and D4 sometimes refers only to specific groups within 12 to 16 (e.g. 14v or 16a2 + 16b1 or 8p, 12p, 13) and rarely to all. We will specify the groups involved in the different studies with detailed results.

## Results of randomized controlled trials comparing different types of dissection (D1 vs. D2, D1 vs. D3, D2 vs. D3)

The full set of randomized trials is listed in Table 2 [6–12, 23–36]. There is a Cochrane meta-analysis published in 2015 that includes long-term oncological follow-up data and postoperative mortality data, including 2515 patients from 8 trials [37]. These studies compared D1 versus D2 dissection [8–12, 23]. It’s worth noting that the Taiwanese study comparing D1 vs. D3 dissection (D2 + 13 + 14v without systematic SPC) was included in the D1 vs. D2 comparison and initially introduced heterogeneity between studies, as it was the only study to show a positive impact on overall survival [28]. Of the trials comparing D2 vs. D3 dissection, two compared D2 dissection with D2 extended to group 16 (16a2 + 16b1) [6, 32]. The last trial compared D3 dissection, including groups 12, 13 and 14, to D4 dissection with the addition of group 16 [33]. Two older trials that did not report survival rates were excluded from this meta-analysis [23, 31]. Their results are detailed below.

**Table 2:** Randomized controlled trials (RCT) comparing different types of lymphadenectomy: morbidity, mortality and long-term survival

	Nb. of patients		Morbidity (%)			Mortality (%)			n-years OS (%)		
**RCT D1 vs. D2**	**D1**	**D2**	**D1**	**D2**	***p*** **-value**	**D1**	**D2**	***p*** **-value**	**D1**	**D2**	***p*** **-value**
Dent et al. [23]	21	22	14	36		0	0		19 (5y)	23 (5y)	
Robertson et al. [24]	25	30	0	80		0	3		72 (5y)	53 (5y)	
Bonenkamp et al. [8, 25]	380	331	25	43	< 0.001	4	10	0.04	45 (5y)	47 (5y)	0.99
									30 (11y)	35 (11y)	0.53
									*LN dissection without SPC sub-group*		
									21 (15y)	35 (15y)	0.03
Cuschieri et al [9, 26]	200	200	28	46	< 0.001	6.5	13	0.04	35 (5y)	35 (5y)	0.43
Degiuli et al [10–12]	133	134	12	17.9	0.178	3	2.2	0.722	66.5 (5y)	64.2 (5y)	0.695
									51.3 (15y)	46.8 (15y)	0.31
									*Sub-group T2-T4N+*		
									38 (5y)	59 (5y)	0.055
									29.4 (15y)	51.4 (15y)	0.035
**RCT D1 + vs. D2**	**D1+**	**D2**	**D1+**	**D2**	***p*** **-value**	**D1+**	**D2**	***p*** **-value**	**D1+**	**D2**	***p*** **-value**
Galizia et al. [27]	36	37	19.4	48.6	0.0172	0	5.4		49.6 (5y)	52.8 (5y)*	0.9068
**RCT D1 vs. D3**	**D1**	**D3**	**D1**	**D3**	***p*** **-value**	**D1**	**D3**	***p*** **-value**	**D1**	**D3**	***p*** **-value**
Wu et al. [28, 29]	110	111	17.1	7.3	0.012	0	0		59.5 (5y)	53.3 (5y)	0.041
**RCT D2 vs. D2+**	**D2**	**D2+**	**D2**	**D2+**	***p*** **-value**	**D2**	**D2+**	***p*** **-value**	**D2**	**D2+**	***p*** **-value**
Yu et al. [30]	32	32	9	28	0.785	NP	NP		71.4 (3y)	65.5 (3y)	0.613
Kulig et al. [31]	141	134	27.7	21.6	0.248	4.9	2.2	0.376	NP	NP	
Yonemura et al. [32]	135	134	22	48		0.7	3.7		52.6 (5y)	55 (5y)	0.801
Sasako et al. [6, 33]	263	260	20.9	28.1	0.07	0	0		69.2 (5y)	70.3 (5y)	0.85
**RCT D3 vs. D4**	**D3**	**D4**	**D3**	**D4**	***p*** **-value**	**D3**	**D4**	***p*** **-value**	**D3**	**D4**	***p*** **-value**
Maeta et al. [34]	35	35	25.7	40	ns	2.8	2.8	ns	38.4 (5y)	52.8 (5y)	0.4238
**RCT D2 vs. D2 + splenectomy (S)**	**D2**	**D2 + S**	**D2**	**D2 + S**	***p*** **-value**	**D2**	**D2 + S**	***p*** **-value**	**D2**	**D2 + S**	***p*** **-value**
Csendes et al. [35]	97	90	NP	NP		3.1	4.4	> 0.7	36 (5y)	42 (5y)	> 0.5
Yu et al. [36]	103	104	8.7	15.4		1	1.9	1	50 (5y)	55 (5y)	> 0.05
Sano et al. [7]	251	254	16.7	30.3	0.0004	0.8	0.4	0.62	76.4 (5y)	75.1 (5y)	0.025

OS: Overall Survival; LN: lymph node; SPC: Splenopancreatectomy; NP not precised; * Disease free survival (DFS)

### Associated morbidity and mortality of different types of dissection

#### D1 vs. D2 (or D3) LN dissection

We have the results of 7 randomized trials [8–12, 23–26].

The South African study by Dent was the first randomized trial to compare D1 dissection with D2 dissection (including excision of the upper mesocolon and pancreatic capsule without mentioning the SPC). No statistical hypothesis was stated, and 43 patients out of 408 who underwent surgery were randomized, with a 3-year follow-up that was purely clinical. The authors concluded that there were more perioperative complications (duration of surgery and transfusion) and postoperative complications (reintervention and length of stay) in the D2 group [23]. The Robertson study compared R3 dissection (D2 dissection with systematic SPC and group 12) (*n* = 30) with R1 dissection (*n* = 25) [24]. There was only one death in the R3 group due to intra-abdominal sepsis. There were no major complications in the R1 group, whereas 47% had subphrenic abscesses, 23% required reoperation and 10% had fistulas in the R3 group. These two studies clearly favoured the D1 dissection in terms of post-operative morbidity.

Subsequently, three large multicentre Western trials compared D1 and D2 dissection: a trial from MRC involving 400 patient [9, 26], a Dutch trial involving 721 patients from 80 hospitals [8, 13, 25], and an Italian trial involving 267 patients from 6 centres [10–12]. In the first two studies, the authors followed the Japanese authors’ guidelines for dissection closely and received training from them. SPC was routinely performed in total gastrectomy according to the old definition of D2 dissection. The Italian trial was designed in the wake of the other two trials and aimed to assess the benefit of D2 dissection without systematic SPC. In the MRC and Dutch trials [8, 9, 13, 25, 26], D2 dissection increased postoperative morbidity and mortality compared with D1 dissection. A meta-analysis of these two trials confirmed these results, showing that mortality was tripled with D2 dissection, with a relative risk of 2.93 (95% CI 1.45–3.45). The “excess mortality” associated with D2 dissection reported in both trials is attributed by most authors to the learning curve of the surgeons participating in the trials. The number of procedures required to overcome the learning curve was estimated to be 25 [4], which is far higher than the numbers reported in the UK trials (32 surgeons for 400 patients, averaging 12.5 patients per surgeon over 7 years) and the Dutch trials (85 surgeons in 80 hospitals over 4 years, resulting in one resection per surgeon per year). In 2006, a single-centre randomized trial from Taiwan enrolled 221 patients [28, 29] and compared D1 dissection with D3 dissection (D1 + celiac trunk branch + hepatic pedicle + 13 + 14 without group 16). This study was performed with a high-quality methodology. SPC was not routinely performed in cases of total gastrectomy, except when an intraoperative examination was positive for group 1 or 11, which applied to only 12% of patients. Patients requiring SPC for necessity (invasion or large LN masses) were excluded. The results of this study [28, 29] also showed that morbidity was increased with D3 dissection (compared to D1 dissection), including a higher incidence of intra-abdominal infectious complications (8.1% vs. 0%, *p* = 0.008) and a trend towards more anastomotic fistulas (4.5% vs. 0%, *p* = 0.060). However, mortality was zero and identical regardless of the extent of dissection, highlighting the expertise of the team involved. The short-term results of the Italian study, conducted in 6 expert centres, comparing D1 dissection (*n* = 133) with D2 dissection without systematic SPC (*n* = 134), were published in 2010 [11]. In the intention-to-treat analysis, the morbidity rates after D2 dissection and D1 dissection were 17.9% and 12.0%, respectively (*p* = 0.178). The in-hospital mortality rate was 3.0% in the D1 group and 2.2% after D2 dissection (*p* = 0.722). The authors concluded that in specialized centres, the complication rate after D2 dissection without systematic SPC was lower than that reported in previously published Western randomized trials and that it could be considered a safe option in this context.

In a last small study, the authors compared D1 + dissection with standard D2 dissection, including group 12a and systematic splenectomy (*n* = 36 vs. 37 patients) [27]. Surgical complications were significantly more frequent in the D2 group, including 2 postoperative deaths, favouring D1 + dissection [27].

The results of the meta-analysis on post-operative mortality are clearly against D2 dissection with an odds ratio of 2.02 [1.34;3.04], considering mortality rates of 3.9% and 7.8%, resulting in 38 additional deaths per 1000 patients operated on with D2 dissection instead of D1 [37]. However, analysis of the results of studies that have evaluated the role of SPC as a primary objective or as a post hoc analysis in this excess mortality is clear. These results, together with the morbidity findings of the Italian study, explain the recent changes in the definitions of dissection discussed above.

#### D2 (or D3) vs. D2 dissection combined with para-aortic LN dissection

Five randomized trials [6, 30–33] have been published. There is considerable variation in the definition of D2 + dissection. It has been referred to as D2+ [30, 31, 33] or D3 [32]. In the most recent study, it was even referred to as D4 and compared to D3 dissection (D2 + 12, 13, 14) vs. D4 = D3 + 16) [33]. It is also worth noting that, with the exception of one study in which splenectomy was performed systematically [32], splenectomy and pancreatectomy were only performed in cases of LN metastases or local invasion of the spleen or pancreas. Of these 5 trials, only one showed a statistically significant increase in morbidity after D2 dissection combined with para-aortic LN dissection (D3) [32]. In the other four trials, morbidity was similar. Finally, postoperative mortality was not increased after lombo-aortic dissection in addition to standard D2 dissection in all 5 trials. These results were consistent with the meta-analysis, which included only 3 of the trials, but concluded that there was no excess postoperative mortality, with no heterogeneity between trials [37].

#### Role of (spleno) pancreatectomy

A total of 6 randomized trials [8, 9, 27, 30, 31, 33] have shown a strong independent association between postoperative morbidity and mortality and resection of the spleen and tail of the pancreas. For example, in the study by Cuschieri et al. [9], the authors demonstrated that morbidity and mortality were significantly higher in cases of splenectomy (59% vs. 22%, *p* < 0.001 and 17% vs. 6%, *p* < 0.001, respectively). These results were confirmed by the Dutch study [8]. Wu et al. [28, 29] also observed an increase in morbidity with splenectomy, but not in mortality. Finally, in other studies [32, 36], pancreatectomy was the most significant predictive factor for postoperative complications and was associated with an increase in morbidity, whether or not LN dissection included para-aortic LN.

#### D2 LN dissection with or without splenectomy

Three studies [7, 35, 36] have been published with conflicting results. These trials compared postoperative morbidity and mortality and survival according to whether or not splenectomy was performed as standard with or without D2 LN dissection during total gastrectomy, excluding patients with splenic continuity invasion and LN metastases requiring mandatory splenectomy. The third study also excluded patients with tumour invasion of the greater curvature (including gastric linitis) [7]. In two studies [7, 35], morbidity was significantly increased with splenectomy (with D2 LN dissection). In the study by Yu et al. [36], morbidity was not significantly affected by splenectomy. Mortality was similar in all three studies [7, 35, 36].

### Oncological outcomes (5-year survival) of different types of lymph node dissection

#### D1 vs. D2 (or D3) LN dissection

In the Dent study, D2 lymphadenectomy did not show a significant benefit in 3-year survival [23]. In the Galizia study comparing D1 + vs. D2 with splenectomy, the primary site of tumour recurrence and 5-year disease-free survival were not different between the two groups. The incidence of involved LN in the additional resection groups was 5%. These results favoured D1 + lymphadenectomy [27]. In the Robertson trial, survival was even better in the R1 group (1511 days vs. 922, *p* < 0.05) [24]. The British and Dutch studies [25, 26] showed no survival benefit for D2 lymphadenectomy compared to D1. However, it was mainly postoperative mortality that negatively affected survival in both trials. In 2010, the 15-year results of the Dutch study were published [13]. D2 lymphadenectomy was ultimately associated with a reduction in locoregional recurrence and cancer-related mortality compared with patients who underwent D1 lymphadenectomy (*p* = 0.01). Overall survival (OS) was significantly lower in patients who underwent splenectomy and pancreatectomy in both the D1 and D2 arms. Subgroup analysis of patients who did not undergo pancreatectomy or splenectomy showed a significantly higher 15-year OS in the D2 group (35% vs. 22%), leading the authors to recommend D2 without systematic SPC lymphadenectomy for resectable gastric cancer. The 5-year results of the Italian trial showed that OS and disease-specific survival (DSS) were 66.5% and 71% after D1 lymphadenectomy and 64.2% and 72.6% after D2 without systematic SPC lymphadenectomy, with no significant difference between the two groups (OS *p* = 0.695, DSS *p* = 0.916) [10]. However, there was significant contamination in the D1 group. Furthermore, subgroup analysis showed a trend towards a benefit of D2 dissection in patients with locally advanced gastric cancer > T1 (DSS 55% for D1 vs. 69% for D2 with *p* = 0.143) and in N + patients (OS rate of 35% for D1 vs. 51% for D2 and DSS rate of 38% for D1 vs. 59% for D2) and in patients with T2-T4 and N+. The long-term results of this trial (15 years), published in 2021, confirmed the absence of a significant difference in OS and DSS between the two groups in the overall population [12]. Subgroup analysis showed a significantly higher DSS in the D2 without systematic SPC lymphadenectomy group in patients with locally advanced gastric cancer > pT1N+ (29.4% vs. 51.4%, *p* = 0.035), confirming the benefit of D2 without systematic SPC lymphadenectomy in these patients. Conversely, DSS was significantly better after D1 lymphadenectomy in early-stage patients and those over 70 years of age (*p* = 0.001) [12].

Only the Taiwanese study, in which postoperative mortality was zero regardless of the extent of lymphadenectomy, showed for the first time that extensive lymphadenectomy (D3) resulted in significantly improved survival [28, 29]. The Cochrane meta-analysis [37] concluded that there was no evidence that D2 lymphadenectomy improved survival. The overall relative risk was 0.91 (95% CI 0,71 − 1,17).

#### D2 (or D3) vs. D2 dissection combined with para-aortic LN dissection

In the most recent meta-analysis, no benefit of D2 + lymphadenectomy was observed (hazard ratio 0.99 [0.81;1.21]) [37]. Since this meta-analysis, a small Chinese randomised trial published in 2019 (70 patients enrolled and 64 analyzed) compared D2 lymphadenectomy with D2+ (D2 with dissection of groups 12b, 8p, 13 and 14v) after open distal gastrectomy. Their primary objective was the “safety” of the procedure, without a clear definition of safety, and no differences were found in terms of complications and long-term outcomes at 3 years, except in the subgroup of patients with duodenal invasion or station n°6 involvement [30].

#### D2 LN dissection with or without splenectomy

The first two trials [35, 36] showed that the number of LN removed was similar whether or not splenectomy was performed (in one of the trials group 10 lymphadenectomy was performed in both groups [36] and in the other trial it was not performed in the group without splenectomy [35]). In the most recent trial published by Sano et al. [7], there was a significant difference between the two groups (64 vs. 59, *p* = 0.005), but only 23% of patients in the no splenectomy group had a group 10 lymphadenectomy or picking (not routinely recommended unless easily accessible). These three trials also showed that 5-year survival was the same whether or not splenectomy was performed as part of a D2 lymphadenectomy. The third trial was the most powerful and was designed as a non-inferiority trial, formally demonstrating the lack of benefit of routine splenectomy in cases of proximal tumours that do not invade the greater curvature [7]. Therefore, it does not appear necessary to perform splenectomy in cases of D2 lymphadenectomy.

## Special case of early gastric cancer

Early gastric cancer (EGC) is defined as a tumour confined to the mucosal and submucosal layers of the stomach wall, regardless of LN involvement. Similar to invasive gastric cancer, LN involvement is an important prognostic factor in EGC. In intramucosal cancers, the risk of metastasis is estimated to be 4% [4]. However, in all series, LN metastases were limited to the first LN group, known as N1 [4]. In contrast, for submucosal cancers, the risk of metastasis is estimated to be 19–23% [4], and metastases can potentially involve any LN group, sometimes even skipping certain nodes [4]. No randomized trials have evaluated LN dissection in EGC. Specific recommendations are given in the conclusion. In cases of cN+ (clinically positive LN), the recommendations are the same as for advanced tumours.

## Controversial issues

### Lymphadenectomy beyond D2

Super-extended LND beyond D2 remains a controversial issue that needs to be discussed. According to Japanese guidelines, proximal gastric cancer invading the greater curvature or with metastatic nodes of the greater curvature requires splenectomy or D2 plus group 10, and distal gastric cancer invading the duodenum requires D2 LND plus group 13. In Western practice, D2 + is only suggested in the setting of conversion surgery, in experimental settings and after neoadjuvant therapy (chemotherapy combined with immunotherapy or targeted therapy according to CPS and HER2 status). The JCOG9501 phase III trial comparing D2 and D2 plus para-aortic nodal dissection did not showed a survival benefit of prophylactic extended lymphadenectomy in the paraaortic area (group 16), but the mortality rate was quite low in both procedures. An approach involving splenic hilar nodal dissection without splenectomy has been developed [38]. Active trials are underway to answer this controversial question: the Indian ELANCE trial (NCT02139605: role of D2 vs. D3 after neoadjuvant treatment in non-metastatic gastric cancer), the Italian Neo-D2-plus trial (NCT03961373: D2 vs. D2 plus in stage IIA-IIIc after neoadjuvant treatment) and the Korean 14VIGTORY (NCT03264807: D2 vs. D2 plus 14v station in T3N + and T4N + gastric cancer). Overall, there is probably a place for such super-extended LND beyond D2 in selected cases and potentially high-risk patients (advanced cT2-T4 forms, patients in good general conditions and under 75 years of age). Tailored D2 + lymphadenectomy may present a viable solution. The utilization of indocyanine green (ICG)-guided LND for locally advanced gastric cancer appears to hold promise [39].

### Extent of LND in curative intended surgery for oligometastatic disease

Regarding surgical management for patients with limited metastatic disease, the AIO-FLOT-3 trial [40] demonstrated the feasibility of such management, with an increase in median overall survival to 31.3 months in oligometastatic patients who underwent surgery with simultaneous resection of the primary tumour and metastatic site after neoadjuvant treatment, compared with 15.9 months in patients who did not receive surgery. In this study, oligometastatic spread was defined as invasion of abdominal, retroperitoneal lymph node metastases only (e.g., para-aortic, intra-aortic-caval, peripancreatic or mesenteric lymph nodes) or 1 unresectable organ site with or without retroperitoneal lymph node metastases. Total or subtotal distal gastrectomy with D2 lymphadenectomy was performed. Patients with lymph node involvement classified as distant metastases (e.g. paraaortic, paracaval or mesenteric lymph nodes), underwent extended lymphadenectomy (one-stage resection) at the intial surgery. Two multicentre randomized trials are ongoing. The RENAISSANCE AIO-FLOT5 phase III multi-centre trial [41] is evaluating the effect of surgical treatment of oligometastatic patients versus systemic therapy alone in limited metastatic gastric and gastro-oesophageal junction cancers. The French SURGIGAST trial [42] compares continuation of chemotherapy with surgical removal of the primary tumour and treatment of the metastatic site followed by chemotherapy in patients with oligometastatic gastric cancer. In both trials, retroperitoneal LN (para-aortal, intra-aorto-caval, para-pancreatic or mesenteric LN) are defined as metastatic.

Overall, there is no clear consensus on the extent of LND in these patients with limited resectable metastatic disease. In clinical practice and in ongoing randomized trials, (total/subtotal) gastrectomy and D2 LND is usually performed, combined with tailored curative treatment of lymph node sites with metastatic appearance during initial extension workup, including group 12 to 16. It should be noted that this conversion surgery is only performed in patients with an excellent response to preoperative systemic chemotherapy and/or targeted therapy.

## Summary of recommendations

The Japanese guidelines advocate performing a D2 lymphadenectomy for any potentially curable cT2-T4 or cT1N + gastric tumour. Group 10 resection by splenectomy should be considered for curable cT2-T4 lesions in the upper part of the stomach invading the greater curvature. For EGC, in addition to the option of a different type of resection (proximal gastrectomy or pylorus-preserving gastrectomy), D1 lymphadenectomy is recommended for cT1aN0 tumours not amenable to endoscopic treatment and for cT1bN0 well-differentiated tumours less than 1.5 cm in diameter. Otherwise, D1 + lymphadenectomy is recommended [20].

According to the NCCN guidelines, for cT1b-T4 tumours, a modified D1 or D2 lymphadenectomy should be performed with sampling of at least 15 LN. Modified D2 lymphadenectomy should only be performed in a high-volume centre by experienced surgeons. Routine or prophylactic resection of the pancreas or spleen is not recommended unless there is T4 tumour invasion of the pancreas or splenic hilum. Splenectomy is acceptable if there is LN involvement at the splenic hilum [19]. The 2013 NCCN guidelines mention D1 + or modified D2 lymphadenectomy in the summary, but only D1 or modified D2 lymphadenectomy in the rationale.

According to the ESMO guidelines [18], patients in Western countries should undergo D2 lymphadenectomy in a high-volume centre with appropriate surgical expertise [Level I, Grade B]. These recommendations highlight the possibility, for EGC, of limiting lymphadenectomy to the first relay of perigastric nodes, based on tumour location, associated of local N2 groups (D1+). The use of sentinel LN may modify these practices.

In France, D2 lymphadenectomy without splenectomy (D1 + lymphadenectomy for nodes 7, 8, 9 and, in the case of proximal gastrectomy, 10 without splenectomy) is recommended [21, 22]. At least 15 LN should be included (expert consensus). For lymphadenectomy in tumours of greater curvature suspected to be T3 or T4, splenectomy should be discussed (expert consensus). This is the only potential indication for splenectomy other than direct invasion. For stage 1 tumours, lymphadenectomy beyond D1 is not recommended as it is very likely to be unnecessary (there is never N2 LN metastasis). D1 lymphadenectomy is also recommended for patients at high surgical risk and for prophylactic gastrectomy in patients with a constitutional CDH1 mutation. All these recommendations are summarized in Table 3.

**Table 3:** Indications for lymphadenectomy based on japanese, european, american and french recommendations

Lymph Node Dissection	JGCA 2021 [20]	ESMO 2022 [18]	NCCN 2019 [19]	TNCD 2022 [22]
**D1** recommendation			**Grade 2 A**	
indications	T1aN0 T1bN0, differentiated, < 1.5 cm	Not recommended	Localized resectable cancer	T1aN0 High operative risk patients Prophylactic gastrectomy for CDH1 mutation
**D1+** recommendation				
indications	T1N0 not mentioned above	T1N0	Not mentioned	
**D2** recommendation		**Grade 2B**	**Grade 2 A**	
indications	T2-T4, T1N+	T2-T4 ou N+ by experienced surgeon	T1b-T4 by experienced surgeon	T1b-T4 ou N+
**D2+** recommendation				
indications	Metastasis to N°10, 14v, 13, 16 lymph node	Not mentioned	Not mentioned	Not mentioned
**Splenectomy** recommendation				
indications	Greater curvature tumor (expert consensus)	Not mentioned	Not recommended	T3-T4 tumor of the greater curvature (*expert consensus*)

JGCA : Japanese Gastric Cancer Association, ESMO : European Society for Medical Oncology, NCCN : National Comprehensive Cancer Network, TNCD : French National Thesaurus of Digestive Oncology

## Conclusion

This review provides a comprehensive overview of the historical context, definitions, and outcomes associated with various lymph node dissection approaches in gastric cancer, offering valuable insights for clinical practice. Over the past 40 years, the extent of LN dissection has been controversial, with historical differences between Asian and Western practices. Recent randomised trials in Asia have led to a reduction in the recommended extent of LN dissection without compromising survival. Western trials have shown no overall survival benefit for D2 dissection, but have associated it with excess morbidity and mortality, particularly in relation to splenectomy or pancreatectomy. European guidelines now recommend modified D2 dissection without systematic splenopancreatectomy in high-volume centres. The article highlights the standardised approach to LN dissection, reflecting a convergence between Western and Asian practices since 2016. The recommendations support the expansion of LN dissection based on tumour stage and type, promoting a nuanced and tailored approach.

## Data Availability

No datasets were generated or analysed during the current study.

## References

[1] Smyth EC, Fassan M, Cunningham D, Allum WH, Okines AFC, Lampis A (2016). Effect of Pathologic Tumor Response and nodal status on Survival in the Medical Research Council adjuvant gastric Infusional Chemotherapy Trial. J Clin Oncol off J Am Soc Clin Oncol.

[2] Amin MB, Edge S, Greene F (2017). Stomach cancer. AJCC Cancer Staging Manual.

[3] Smith DD, Schwarz RR, Schwarz RE (2005). Impact of Total Lymph Node Count on staging and Survival after Gastrectomy for gastric Cancer: data from a large US-Population database. J Clin Oncol.

[4] Mariette C, Piessen G, Vons C (2008). La Chirurgie ganglionnaire dans les cancers de l’œsophage et de l’estomac. J Chir (Paris).

[5] Zhou Y, Yu F, Wu L, Ye F, Zhang L, Li Y (2015). Survival after Gastrectomy in Node-negative gastric Cancer: a review and Meta-analysis of prognostic factors. Med Sci Monit Int Med J Exp Clin Res.

[6] Sasako M, Sano T, Yamamoto S, Kurokawa Y, Nashimoto A, Kurita A (2008). D2 lymphadenectomy alone or with para-aortic nodal dissection for gastric cancer. N Engl J Med.

[7] Sano T, Sasako M, Mizusawa J, Yamamoto S, Katai H, Yoshikawa T (2017). Randomized Controlled Trial to evaluate splenectomy in total gastrectomy for proximal gastric carcinoma. Ann Surg.

[8] Bonenkamp JJ, Hermans J, Sasako M, van de Velde CJ, Welvaart K, Songun I (1999). Extended lymph-node dissection for gastric cancer. N Engl J Med.

[9] Cuschieri A, Weeden S, Fielding J, Bancewicz J, Craven J, Joypaul V (1999). Patient survival after D1 and D2 resections for gastric cancer: long-term results of the MRC randomized surgical trial. Surgical co-operative group. Br J Cancer.

[10] Degiuli M, Sasako M, Ponti A, Vendrame A, Tomatis M, Mazza C (2014). Randomized clinical trial comparing survival after D1 or D2 gastrectomy for gastric cancer. Br J Surg.

[11] Degiuli M, Sasako M, Ponti A, Italian Gastric Cancer Study Group (2010). Morbidity and mortality in the Italian gastric Cancer Study Group randomized clinical trial of D1 versus D2 resection for gastric cancer. Br J Surg.

[12] Degiuli M, Reddavid R, Tomatis M, Ponti A, Morino M, Sasako M et al. D2 dissection improves disease-specific survival in advanced gastric cancer patients: 15-year follow-up results of the Italian gastric cancer study group D1 versus D2 randomised controlled trial. Eur J Cancer Oxf Engl. 1990. 2021;150:10–22. 10.1016/j.ejca.2021.03.031 33887514

[13] Songun I, Putter H, Kranenbarg EMK, Sasako M, van de Velde CJH (2010). Surgical treatment of gastric cancer: 15-year follow-up results of the randomised nationwide Dutch D1D2 trial. Lancet Oncol.

[14] Smyth EC, Verheij M, Allum W, Cunningham D, Cervantes A, Arnold D (2016). Gastric cancer: ESMO Clinical Practice guidelines for diagnosis, treatment and follow-up. Ann Oncol off J Eur Soc Med Oncol.

[15] Kajitani T (1981). The general rules for the gastric cancer study in surgery and pathology. Part I. Clinical classification. Jpn J Surg.

[16] Japanese Gastric Cancer Association (2011). Japanese gastric cancer treatment guidelines 2010 (ver. 3). Gastric Cancer off J Int Gastric Cancer Assoc Jpn Gastric Cancer Assoc.

[17] Sano T, Aiko T (2011). New Japanese classifications and treatment guidelines for gastric cancer: revision concepts and major revised points. Gastric Cancer off J Int Gastric Cancer Assoc Jpn Gastric Cancer Assoc.

[18] Lordick F, Carneiro F, Cascinu S, Fleitas T, Haustermans K, Piessen G (2022). Gastric cancer: ESMO Clinical Practice Guideline for diagnosis, treatment and follow-up. Ann Oncol off J Eur Soc Med Oncol.

[19] Ajani JA, D’Amico TA, Bentrem DJ, Chao J, Cooke D, Corvera C (2022). Gastric Cancer, Version 2.2022, NCCN Clinical Practice guidelines in Oncology. J Natl Compr Cancer Netw JNCCN.

[20] Japanese Gastric Cancer Association (2023). Japanese gastric Cancer Treatment guidelines 2021 (6th edition). Gastric Cancer off J Int Gastric Cancer Assoc Jpn Gastric Cancer Assoc.

[21] Slim K, Blay JY, Brouquet A, Chatelain D, Comy M, Delpero JR (2009). [Digestive oncology: surgical practices]. J Chir (Paris).

[22] Zaanan A, Barret M, Buecher B, Benhaim L, Chapelle N, Dubreuil O, Ducreux M, Durand-Labrunie J, Fares N, Gagniere J, Granger V, Ernst O, Renaud F, Vendrely V, Michel P. M. Ducreux, O. Bouché. « cancer de l’estomac ». Thésaurus national de cancérologie Digestive, octobre 2022, en ligne [http://www.tncd.org]

[23] Dent DM, Madden MV, Price SK (1988). Randomized comparison of R1 and R2 gastrectomy for gastric carcinoma. Br J Surg.

[24] Robertson CS, Chung SC, Woods SD, Griffin SM, Raimes SA, Lau JT (1994). A prospective randomized trial comparing R1 subtotal gastrectomy with R3 total gastrectomy for antral cancer. Ann Surg.

[25] Bonenkamp JJ, Songun I, Hermans J, Sasako M, Welvaart K, Plukker JT (1995). Randomised comparison of morbidity after D1 and D2 dissection for gastric cancer in 996 Dutch patients. Lancet Lond Engl.

[26] Cuschieri A, Fayers P, Fielding J, Craven J, Bancewicz J, Joypaul V (1996). Postoperative morbidity and mortality after D1 and D2 resections for gastric cancer: preliminary results of the MRC randomised controlled surgical trial. The Surgical Cooperative Group. Lancet Lond Engl.

[27] Galizia G, Lieto E, De Vita F, Castellano P, Ferraraccio F, Zamboli A (2015). Modified versus standard D2 lymphadenectomy in total gastrectomy for nonjunctional gastric carcinoma with lymph node metastasis. Surgery.

[28] Wu CW, Hsiung CA, Lo SS, Hsieh MC, Shia LT, Whang-Peng J (2004). Randomized clinical trial of morbidity after D1 and D3 surgery for gastric cancer. Br J Surg.

[29] Wu CW, Hsiung CA, Lo SS, Hsieh MC, Chen JH, Li AFY (2006). Nodal dissection for patients with gastric cancer: a randomised controlled trial. Lancet Oncol.

[30] Yu P, Du Y, Xu Z, Huang L, Cheng X (2019). Comparison of D2 and D2 plus radical surgery for advanced distal gastric cancer: a randomized controlled study. World J Surg Oncol.

[31] Kulig J, Popiela T, Kolodziejczyk P, Sierzega M, Szczepanik A, Polish Gastric Cancer Study Group (2007). Standard D2 versus extended D2 (D2+) lymphadenectomy for gastric cancer: an interim safety analysis of a multicenter, randomized, clinical trial. Am J Surg.

[32] Yonemura Y, Wu CC, Fukushima N, Honda I, Bandou E, Kawamura T (2006). Operative morbidity and mortality after D2 and D4 extended dissection for advanced gastric cancer: a prospective randomized trial conducted by Asian surgeons. Hepatogastroenterology.

[33] Sano T, Sasako M, Yamamoto S, Nashimoto A, Kurita A, Hiratsuka M (2004). Gastric cancer surgery: morbidity and mortality results from a prospective randomized controlled trial comparing D2 and extended para-aortic lymphadenectomy–Japan Clinical Oncology Group study 9501. J Clin Oncol off J Am Soc Clin Oncol.

[34] Maeta M, Yamashiro H, Saito H, Katano K, Kondo A, Tsujitani S (1999). A prospective pilot study of extended (D3) and superextended para-aortic lymphadenectomy (D4) in patients with T3 or T4 gastric cancer managed by total gastrectomy. Surgery.

[35] Csendes A, Burdiles P, Rojas J, Braghetto I, Diaz JC, Maluenda F (2002). A prospective randomized study comparing D2 total gastrectomy versus D2 total gastrectomy plus splenectomy in 187 patients with gastric carcinoma. Surgery.

[36] Yu W, Choi GS, Chung HY (2006). Randomized clinical trial of splenectomy versus splenic preservation in patients with proximal gastric cancer. Br J Surg.

[37] Mocellin S, McCulloch P, Kazi H, Gama-Rodrigues JJ, Yuan Y, Nitti D (2015). Extent of lymph node dissection for adenocarcinoma of the stomach. Cochrane Database Syst Rev.

[38] Faiz Z, Hayashi T, Yoshikawa T (2021). Lymph node dissection for gastric cancer: establishment of D2 and the current position of splenectomy in Europe and Japan. Eur J Surg Oncol J Eur Soc Surg Oncol Br Assoc Surg Oncol.

[39] Huang ZN, Tang YH, Zhong Q, Li P, Xie JW, Wang JB et al. Assessment of laparoscopic indocyanine green tracer-guided lymphadenectomy after neoadjuvant chemotherapy for locally advanced gastric cancer: a randomized controlled trial. Ann Surg. 2024. 10.1097/SLA.0000000000006242 38375670

[40] Al-Batran SE, Homann N, Pauligk C, Illerhaus G, Martens UM, Stoehlmacher J (2017). Effect of Neoadjuvant Chemotherapy followed by Surgical Resection on Survival in patients with Limited Metastatic gastric or gastroesophageal Junction Cancer: the AIO-FLOT3 Trial. JAMA Oncol.

[41] Al-Batran SE, Goetze TO, Mueller DW, Vogel A, Winkler M, Lorenzen S (2017). The RENAISSANCE (AIO-FLOT5) trial: effect of chemotherapy alone vs. chemotherapy followed by surgical resection on survival and quality of life in patients with limited-metastatic adenocarcinoma of the stomach or esophagogastric junction - a phase III trial of the German AIO/CAO-V/CAOGI. BMC Cancer.

[42] University Hospital. Lille. Surgical Resection Plus Chemotherapy Versus Chemotherapy Alone in Oligometastatic Stage IV Gastric Cancer - a Multicenter, Prospective, Open-labeled, Two-armed, Randomized, Controlled Phase III Trial. clinicaltrials.gov; 2022 Mar [cited 2024 Jan 1]. Report No.: NCT03042169. https://clinicaltrials.gov/study/NCT03042169

